# The Relationship Between Psychological Stress and Anxiety with Gastrointestinal Symptoms Before and During a 56 km Ultramarathon Running Race

**DOI:** 10.1186/s40798-021-00389-5

**Published:** 2021-12-11

**Authors:** Charles S. Urwin, Luana C. Main, Antonina Mikocka-Walus, David R. Skvarc, Spencer S. H. Roberts, Dominique Condo, Amelia J. Carr, Lilia Convit, William Jardine, Shant S. Rahman, Rhiannon M. J. Snipe

**Affiliations:** 1 Centre for Sport Research, School of Exercise and Nutrition Sciences, Deakin University, Melbourne, VIC Australia; 2 Institute for Physical Activity and Nutrition, School of Exercise and Nutrition Sciences, Deakin University, Melbourne, VIC Australia; 3 Centre for Social & Early Emotional Development, School of Psychology, Deakin University, Melbourne, VIC Australia

**Keywords:** Endurance exercise, Competitive stress, Competitive anxiety, Gastrointestinal discomfort

## Abstract

**Background:**

This study assessed relationships and sex differences between psychological state (recovery, stress, anxiety, and self-confidence) and gastrointestinal symptoms (GIS) prior to and during a 56 km ultramarathon running race and identified predictive factors of race GIS. Forty-four (26 males, 18 females) ultramarathon competitors completed anxiety, recovery, stress and GIS questionnaires for three days prior to the race and immediately pre-race. Race GIS were assessed immediately post-race. Spearman’s rank order, Mann–Whitney *U* tests and regression analyses were used to determine correlations and identify sex differences between psychological state and GIS and determine predictors of race GIS.

**Results:**

Race GIS were significantly correlated with recovery (*r*_s_ =  − 0.381, *p* = 0.011), stress (*r*_s_ = 0.500, *p* = 0.001) and anxiety (*r*_s_ = 0.408, *p* = 0.006), calculated as the mean of the three days preceding the race and on race morning. The correlation between anxiety and GIS was strongest in the 24 h immediately prior to the race (all *r*_s_ > 0.400, and all *p* < 0.05), but unclear patterns were identified for stress and recovery. Regression analyses showed 36% and 40% of variation in the severity and number of race GIS was accounted for by body mass and measures of stress, anxiety, and GIS over the three days preceding the race and on race morning (both *p* < 0.001). There were no sex differences in the number and severity of GIS leading up to or during the race (all *p* > 0.05), however, females reported greater state anxiety (*p* = 0.018) and lower self-confidence than males (*p* = 0.006) over the three days preceding the race and on race morning.

**Conclusion:**

Endurance athletes that experience GIS during competition should investigate elevated stress and/or anxiety as a potential contributor and identify if management strategies can reduce the occurrence and severity of GIS.

**Supplementary Information:**

The online version contains supplementary material available at 10.1186/s40798-021-00389-5.

## Key Points

- Endurance athletes should monitor stress, anxiety and GIS using reliable and valid questionnaires over the three days prior to competition.
- If GIS occur alongside elevated anxiety and/or stress, athletes may consider the implementation of low-risk anxiety-reduction strategies, although more evidence is required to establish their efficacy in a competition context.
- If GIS occur in the absence of elevated stress and/or anxiety or if athletes present with a history of prior GIS, consider medical pathophysiology and/or nutritional and exercise-stress related causes.

## Background

Gastrointestinal symptoms (GIS) are frequently reported by endurance athletes, with up to 96% of ultramarathon runners reporting GIS during their event [1, 2]. The occurrence of GIS can be deleterious to endurance exercise performance and can result in withdrawal from competition [3, 4]. Therefore, identifying contributors to GIS and developing strategies to alleviate or avoid them are of interest to athletes, coaches and sports practitioners. Current research suggests that GIS occur more frequently with increased exercise intensity, prolonged exercise duration [1], and running exercise (rather than cycling, for example) [4]. The GIS associated with the physiological responses to these exercise characteristics have been reviewed in detail by Costa et al. [1]. However, identifying the aetiology of exercise-associated GIS is complex due to a multi-factorial causality [1, 4, 5]. Other potential contributors to GIS include nutrition intake, and participant characteristics such as greater body mass, lesser training/running experience, older age, female sex and a history of prior GIS [6].

Emerging evidence suggests that psychological states, such as elevated stress and anxiety, may also contribute to exercise-associated GIS [5, 7]. Acute and chronic stress and anxiety may contribute to GIS in endurance athletes via the gut-brain axis [5, 8]. Chronic stress and anxiety may also affect GIS via visceral hypersensitivity, a mechanism that has been proposed to contribute to the psychobiological basis of GIS in irritable bowel syndrome (IBS) [5, 7]. Much of the foundational work regarding the interplay between stress, anxiety and exercise-related GIS can be attributed to Wilson and colleagues [5, 9, 10]. Specifically, their laboratory has reported that chronic stress and anxiety have been correlated with GIS (Spearman’s rho 0.29 and 0.27, respectively) in experienced distance runners during a 30-day training period [9]. Wilson et al. have also reported associations between trait and competition anxiety and GIS (Spearman’s rho 0.33 and 0.28, respectively) and increased risk of nausea (adjusted odds ratio 3.44 and 5.14, respectively) and regurgitation/reflux (adjusted odds ratio 3.46 and 4.74, respectively) with higher trait and state-anxiety, but not chronic stress, during endurance sport (running, duathlon or triathlon) competition [10, 11]. Concurrent increases in stress and GIS have also been identified during an army combat training course [12], and in triathletes/multisport athletes [13, 14], although few of these studies performed traditional statistical analyses to explore the relationship between stress and GIS [5]. While existing research supports a link between anxiety and GIS, no research to date has investigated acute pre-competition stress and anxiety using sport-specific tools [15] administered over the days prior to competition and at the competition venue, where acute competition stress and anxiety are likely to be at its peak. It is possible that female endurance athletes may experience greater disturbances in the gut-brain axis than male endurance athletes. Specifically, female athletes are more likely to report experiencing GIS during endurance exercise compared to male athletes [16, 17], though the cause of this potential sex difference is not yet clear [18]. In addition, a recent meta-analysis identified that female athletes tend to report higher anxiety scores when compared to male counterparts [19]. While it appears that female athletes experience greater anxiety and GIS than male athletes, it is not yet clear whether the strength of the relationship between psychological state (both stress and anxiety) and GIS differs by sex in the context of endurance exercise. Identifying potential sex differences in the relationship between psychological state and GIS will improve the identification and implementation of appropriate strategies to minimise GIS during endurance exercise.

Considering the current dearth of research on acute competitive stress and anxiety with GIS in athletes, the primary aim of this study was to assess the relationship between pre-race psychological state (stress, anxiety, recovery, and self-confidence) and GIS before and during a 56 km ultramarathon running race. Secondary aims included identifying predictive factors for GIS during the ultramarathon race, examining sex differences in psychological state and GIS, and assessing the impact of GIS on ultramarathon running performance.

## Methods

### Study Design, Participants and Recruitment

Runners in the 2020 Two Bays Trail 56 km Ultramarathon running race were identified for recruitment after the Deakin University Human Research Ethics Committee approved all protocols (2019-389). Recruitment was conducted via race-specific emails and social media platforms including Facebook (Menlo Park, California, USA). Runners provided written consent before completing an online screening questionnaire using research electronic data capture (REDCap) [20] to determine eligibility for participation in the study. Inclusion criteria incorporated healthy males and females aged ≥ 18 years who were registered to compete in the race. Individuals were excluded from participation if they presented with pre-existing gastrointestinal conditions such as coeliac disease, inflammatory bowel disease, IBS, functional bowel disorder and/or prior gastrointestinal surgery. Forty-six participants met the inclusion criteria for this study; however, two did not complete the race and their data were subsequently excluded from analysis and the study. A total of 44 runners (26 males, 18 females) completed all data collection and were included in the final analyses.

**Fig. 1:**
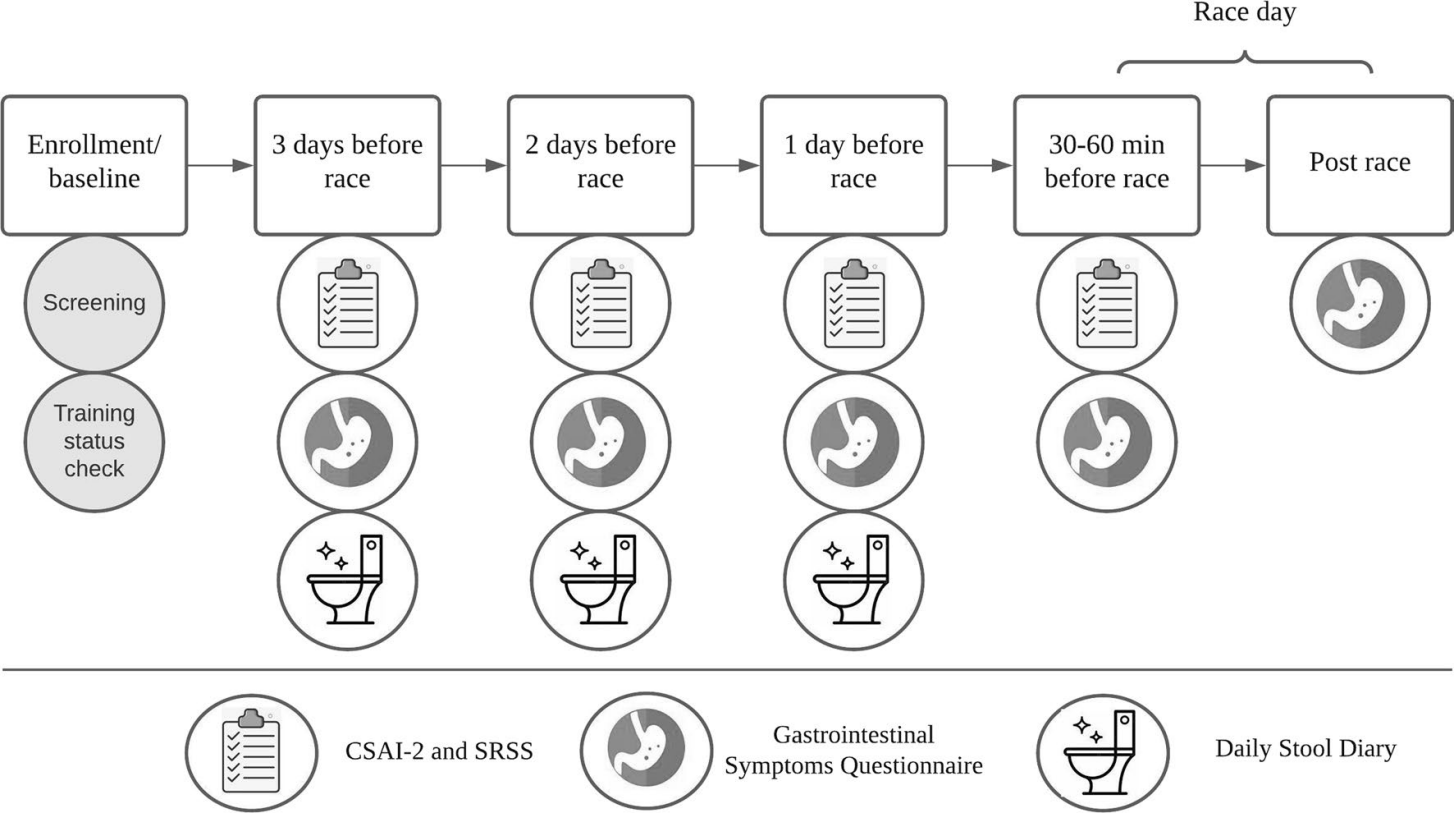
Schematic of the protocols conducted throughout this study. CSAI-2, Competitive State Anxiety Inventory-2; SRSS, Short Recovery and Stress Scale. Screening and training status assessment included the following measurements: height (cm), body mass (kg), age (years), biological sex, training history (years of experience), weekly training volume (hours per week)

This study employed a prospective observational design that allowed participants to consume their habitual diet and exercise training. Data for this study were collected at enrolment into the study (i.e. participant characteristics), over the three days before the race (i.e. three days before the race = T-3, two days before the race = T-2, one day before the race = T-1) and within 60 min pre (T0)- and post-race (TPost) on race day as per Fig. 1. Participant characteristics (self-reported height, body mass, age, training experience) were obtained via an online questionnaire in REDCap. Each evening for the three days prior to the race (T-3, T-2, T-1), participants were emailed a link to an online (REDCap) survey that included the Competitive State Anxiety Inventory-2 (CSAI-2) [21], the Short Recovery and Stress Scale (SRSS) [22], a GIS Questionnaire [23], and a stool diary.

### Questionnaires, Scales and Diaries

The CSAI-2 (Cronbach’s *a* =  ~ 0.87) comprises nine statements each for measures of cognitive state anxiety, somatic state anxiety and self-confidence, which participants respond to on a 4-point Likert scale [21]. Four values were obtained from the CSAI-2; the sum of responses related to cognitive state anxiety, somatic state anxiety and self-confidence, as well as total state anxiety (the sum of cognitive and somatic state anxiety questions).

The SRSS (Cronbach’s *a* =  ~ 0.74) is a psychometrically valid and sport-specific tool that comprises four items each for measures of stress (e.g. muscular stress and overall stress) and recovery (e.g. physical and psychological restorative processes [24]) [22]. The SRSS was selected for its specificity, brevity and validity in athletes [25]. Two values were obtained from the SRSS; the sum of all items related to recovery and the sum of all items related to stress.

A reliable GIS assessment tool was used to measure the incidence and severity of GIS [23]. Participants rated the extent to which they were experiencing 14 GIS (seven for upper GIS, six for lower GIS, and one other symptom) on an 11-point Likert type scale, as previously described [23]. Any GIS that was rated as ≥ 5 out of 10 was classified as ‘severe’. The number (incidence) of GIS reported and number of ‘severe’ GIS reported (both minimum possible value of 0, and maximum possible value of 14), as well as the cumulative severity of all GIS (minimum possible value of 0, maximum possible value of 140), were obtained at each time-point.

A stool diary, based on the valid and reliable Bristol stool chart, was used to identify symptoms of abnormal defecation (e.g. Type 7 stool represents diarrhoea) and was included in the pre-race lower GIS [26]. Approximately 30–60 min prior to race commencement (T0), participants completed the CSAI-2, SRSS, GIS questionnaire and stool diary in hard-copy (paper), and within 10 min of race completion (TPost), participants completed the GIS questionnaire as hard-copy to capture GIS that were experienced during the race.

### Statistical Analyses

Descriptive statistics (mean, 95% CIs) were reported for participant characteristics. The distribution of each variable was assessed using a Shapiro–Wilk test; the majority of variables were identified as not normally distributed (body mass, body height, training experience, number and severity of GIS, cognitive state anxiety, somatic state anxiety and total state anxiety). As such, subsequent analyses were conducted using nonparametric methods. Correlations between variables of psychological state and GIS (both number and severity) over the pre-race period (i.e. T-3, T-2, T-1, and T0 assessed individually) were identified using Spearman’s rank-order tests. Due to the largely unclear or inconsistent patterns identified within these analyses, mean psychological state over the days before the race (T-3, T-2., T-1, and T0) was calculated and used in subsequent analyses as a representation of psychological state in the lead up to competition. Spearman’s rank-order tests were performed to determine the correlation between race GIS (TPost) and pre-race (i.e. mean of T-3, T-2, T-1, T0) recovery, stress, state anxiety (cognitive, somatic and total), and self-confidence, and also between GIS and race performance (time to race completion). Correlations were categorised according to statistical convention [27], whereby a Spearman’s rho (*r*_s_) < 0.3 was weak, an *r*_s_ between 0.3 and 0.6 was moderate, and an *r*_s_ between 0.6 and 0.9 was strong. Regression analyses to predict race GIS (TPost, number and severity) were conducted using variables that significantly correlated with race GIS (TPost) in the prior analyses, as well as those GIS (number and severity according to the targeted prediction) reported before the race (i.e. T-3, T-2, T-1, T0). A square root transformation was performed for mean severity of GIS for the regression analyses, due to data skew. Mann–Whitney *U* tests were used to identify differences between male and female participants for all pre-race variables (i.e. mean of T-3, T-2, T-1, and T0). As some differences were detected between males and females, correlational analyses were performed for all participants as a collective and then for males and females separately. Mann–Whitney *U* tests were also used to identify differences between participants who did and did not report at least one severe GIS (rating ≥ 5) during the race (TPost) in terms of pre-race (T-3, T-2, T-1, T0) recovery, stress, state anxiety (cognitive, somatic and total), and self-confidence, as well as time to race completion. All statistical analyses were conducted using Stata v15, and significance was accepted at an alpha level of *p* < 0.05.

## Results

Time to race completion and self-reported participant characteristics are presented in Table 1. During the race (TPost), participants reported a mean (95% CIs) incidence of 3.2 (2.5, 4.0) GIS out of a possible 14, and a mean severity rating of 11.2 (7.4, 14.9) out of a possible 140 (Table 1). Flatulence (61% of participants) and belching (59% of participants) were reported by the greatest proportion of the runners during the race (TPost, 

Additional file 1: Table S1).

**Table 1:** Gastrointestinal symptoms, recovery, stress, anxiety and participant characteristics of female and male participants in a 56 km ultramarathon running race

	All participants			Females			Males			*p* value
	Mean	95% CIs		Mean	95% CIs		Mean	95% CIs		
*Race performance*										
Time to completion (min)	388.0	372.7	403.3	399.5	379.1	419.9	380.0	357.8	402.3	0.2098
*Gastrointestinal symptoms*										
Number of pre-race GIS	1.4	1.0	1.7	1.7	1.0	2.5	1.1	0.7	1.5	0.0963
Number of race GIS	3.2	2.5	4.0	2.7	1.4	3.9	3.6	1.9	4.5	0.2028
Severity of pre-race GIS	4.2	2.8	5.5	5.7	2.8	8.7	3.1	2.7	4.2	0.0540
Severity of race GIS	11.2	7.4	14.9	9.2	2.3	16.0	12.5	8.0	17.1	0.3776
*Recovery, stress and anxiety*										
Recovery	17.1	16.2	17.9	16.3	15.0	17.5	17.6	16.3	18.9	0.1501
Stress	7.1	6.0	8.2	8.0	6.2	9.8	6.5	5.1	7.9	0.1820
Cognitive state anxiety	14.4	13.0	15.8	16.5	13.8	19.3	12.9	11.8	14.0	**0.0345**
Somatic state anxiety	14.0	12.8	15.3	15.8	13.4	18.1	12.8	11.6	14.0	**0.0423**
Self-confidence	25.1	23.1	27.0	22.0	19.3	24.6	27.2	24.7	29.7	**0.0059**
Total state anxiety	28.4	26.0	30.8	32.3	27.5	37.1	25.7	23.6	27.8	**0.0181**
*Participant characteristics*										
Age (years)	40.3	38.0	42.7	37.6	34.1	41.0	42.2	39.1	45.4	**0.0480**
Body mass (kg)	72.5	68.4	76.5	60.8	58.3	63.3	80.5	76.1	85.0	**0.0000**
Stature (cm)	170.8	162.4	179.2	157.5	138.0	177.0	180.0	177.0	183.0	**0.0000**
Body mass index (kg/m^2^)	23.6	22.8	24.4	21.9	21.0	22.8	24.8	23.9	25.7	**0.0001**
Running experience (years)	5.1	4.1	6.0	4.3	2.9	5.8	5.6	4.2	6.9	0.2124
Training hours per week	4.9	4.4	5.3	4.7	3.9	5.6	5.0	4.4	5.5	0.6201

Number of GIS: mean number of GIS reported from all pre-race times or during the race (minimum of 0, maximum of 14); severity of GIS: mean severity rating of all GIS reported from all pre-race times or during the race (minimum of 0, maximum of 140); recovery: mean recovery score from all pre-race times (minimum of 0, maximum of 24); stress: mean stress score from all pre-race times (minimum of 0, maximum of 24); cognitive state anxiety: mean cognitive state anxiety score from all pre-race times (minimum of 0, maximum of 36); somatic state anxiety: mean somatic state anxiety score from all pre-race times (minimum of 0, maximum of 36); self-confidence: mean self-confidence score from all pre-race times (minimum of 0, maximum of 36); total state anxiety: mean total state anxiety score from all pre-race times (minimum of 0, maximum of 72). Bolded values represent a statistically significant difference between female and male participants (*p* < 0.05)

No differences were detected between males and females for time to race completion or for the incidence or severity of GIS either pre-race or during the race (T-3, T-2, T-1, T0, TPost, all *p* > 0.05, Table 1). Females reported significantly greater cognitive state anxiety (*p* = 0.035), somatic state anxiety (*p* = 0.042) and total state anxiety (*p* = 0.018), and lower self-confidence (*p* = 0.006, Table 1) when compared to males pre-race (T-3, T-2, T-1, T0).

Statistically significant correlations were detected between race GIS (number and severity) with pre-race recovery, stress, state anxiety (cognitive, somatic and total) and body mass for all participants (Table 2). In females, no significant correlations were detected between race GIS (number and severity) and any other variable (Table 2). However, for male participants, statistically significant correlations that were moderate or strong were detected between race GIS (number and severity) with pre-race stress and state anxiety (cognitive, somatic and total), and significant correlations were detected with pre-race recovery and self-confidence (Table 2). A significant but weak positive correlation between race GIS (severity only) and time to race completion was detected for all participants combined and for male participants, but not for female participants (Table 2). Weak and non-significant correlations were detected between age and race GIS (number: *r*_s_ = 0.068, *p* = 0.661; severity: *r*_s_ = 0.062, *p* = 0.691, Table 2).

**Table 2:** Correlative factors for race gastrointestinal symptoms (GIS)

	All participants				Females				Males			
	Number of race GIS		Severity of race GIS		Number of race GIS		Severity of race GIS		Number of race GIS		Severity of race GIS	
	*r*_s_	*p* value	*r*_s_	*p* value	*r*_s_	*p* value	*r*_s_	*p* value	*r*_s_	*p* value	*r*_s_	*p* value
Time to race completion	0.272	0.074	**0.317**	**0.036**	0.449	0.061	0.288	0.247	0.277	0.170	**0.415**	**0.035**
Number of pre-race GIS	**0.423**	**0.004**	**0.357**	**0.017**	0.354	0.150	0.398	0.102	**0.575**	**0.002**	**0.396**	**0.045**
Severity of pre-race GIS	**0.407**	**0.006**	**0.351**	**0.020**	0.432	0.074	**0.485**	**0.041**	**0.480**	**0.013**	0.307	0.127
Recovery	** − 0.381**	**0.011**	** − 0.318**	**0.035**	− 0.309	0.213	− 0.228	0.363	** − 0.516**	**0.007**	** − 0.457**	**0.019**
Stress	**0.500**	**0.001**	**0.470**	**0.001**	0.370	0.131	0.434	0.072	**0.703**	**0.000**	**0.619**	**0.001**
Cognitive state anxiety	**0.410**	**0.006**	**0.303**	**0.046**	0.315	0.204	0.196	0.435	**0.678**	**0.000**	**0.583**	**0.002**
Somatic state anxiety	**0.375**	**0.012**	**0.315**	**0.037**	0.158	0.531	0.120	0.636	**0.710**	**0.000**	**0.662**	**0.000**
Self-confidence	− 0.240	0.117	− 0.169	0.273	− 0.295	0.235	− 0.117	0.644	** − 0.436**	**0.026**	** − 0.417**	**0.034**
Total state anxiety	**0.408**	**0.006**	**0.309**	**0.042**	0.286	0.250	0.175	0.486	**0.747**	**0.000**	**0.666**	**0.000**
Age	0.068	0.661	0.062	0.691	0.110	0.664	0.055	0.829	− 0.061	0.768	− 0.089	0.665
Body mass	**0.307**	**0.043**	**0.326**	**0.031**	− 0.018	0.944	− 0.057	0.824	**0.394**	**0.047**	0.371	0.062
Stature	0.240	0.117	0.134	0.134	− 0.305	0.218	− 0.267	0.285	0.305	0.130	0.129	0.529
Body mass index	0.250	0.102	0.275	0.071	0.208	0.407	0.157	0.535	0.137	0.506	0.164	0.423
Running experience	− 0.093	0.547	− 0.080	0.608	− 0.386	0.113	− 0.238	0.342	− 0.050	0.808	− 0.132	0.520
Training hours per week	− 0.127	0.410	− 0.092	0.554	− 0.075	0.768	− 0.031	0.903	− 0.251	0.217	− 0.232	0.255

*r*_s_: Spearman’s rank correlation coefficient, bolded values represent correlation that was both statistically significant (*p* < 0.05) and *r*_s_ ≥ 0.3, representing ‘moderate’ or stronger correlation. Pre-race GIS (number and severity): mean scores from all pre-race times (T-3, T-2. T-1, T0); Recovery: mean recovery score from all pre-race times; stress: mean stress score from all pre-race times; cognitive state anxiety: mean cognitive state anxiety score from all pre-race times; somatic state anxiety: mean somatic state anxiety score from all pre-race times; self-confidence: mean self-confidence score from all pre-race times; total state anxiety: mean total state anxiety score from all pre-race times. Number of race GIS reported: mean number of symptoms reported by participants during the race (TPost, for all, female or male); severity of race GIS: the mean sum of all GIS severity scores reported by participants during the race (TPost, for all, female or male)

A regression analysis which included pre-race (T-3, T-2, T-1, T0) recovery, stress, total anxiety and prior GIS (number or severity) and body mass statistically significantly predicted 40% of the variability in the number (*p* < 0.001) and 36% of the variability in the severity (*p* < 0.001) of GIS reported during the race (TPost, Table 3, Additional file 1: Table S2). For both number and severity of race GIS (TPost), statistically significant predictive factors were prior GIS (T-3, T-2, T-1, T0) and body mass (all *p* < 0.05, Table 3). Participants that reported at least one severe GIS during the race (TPost rating ≥ 5) reported significantly higher pre-race (T-3, T-2, T-1, T0) stress than those participants who did not report any severe GIS (*p* = 0.049). No differences were detected between participants that reported severe vs non-severe GIS for race performance (*p* = 0.064), pre-race (T-3, T-2, T-1, T0) recovery (*p* = 0.563), cognitive state anxiety (*p* = 0.316), somatic state anxiety (*p* = 0.186), self-confidence (*p* = 0.358) or total anxiety (*p* = 0.220).

**Table 3:** Regression coefficients for predicting gastrointestinal symptoms during a 56 km Ultramarathon running race

Model	Independent variable	Coefficients			Squared semi-partial correlations	*t*	Sig
		Unstandardised *β*	*β*	SE			*p* value
1. Number of race GIS							
	(Constant)	− 4.349		4.053		− 1.07	0.290
	Number of pre-race GIS	0.190	0.388	0.068	0.110	2.81	**0.008**
	Recovery	− 0.007	− 0.036	0.041	0.000	− 0.18	0.862
	Stress	0.046	0.272	0.039	0.019	1.16	0.253
	Total state anxiety	0.003	0.042	0.012	0.001	0.26	0.795
	Body mass	0.074	0.410	0.023	0.150	3.28	**0.002**
2. Severity of race GIS							
	(Constant)	− 4.223		3.2860		− 1.29	0.207
	Severity of pre-race GIS	0.040	0.381	0.015	0.106	2.66	**0.011**
	Recovery	0.018	0.115	0.034	0.005	0.55	0.586
	Stress	0.060	0.451	0.033	0.050	1.83	0.075
	Total state anxiety	− 0.007	− 0.112	0.010	0.007	− 0.67	0.507
	Body mass	0.057	0.396	0.019	0.135	3.00	**0.005**

Number of race GIS: mean number of GIS reported by each participant during the race (TPost); severity of race GIS: mean severity of GIS reported by each participant during the race (TPost). Number of pre-race GIS, recovery, stress, total state anxiety and severity of pre-race GIS were the sum of all times prior to race day (i.T-3, T-2, T-1, T0). Bolded values represents that independent variable contributes statistically significant to the prediction of the dependent variable (*p* < 0.05). A square root transformation was performed for mean severity of GIS to account for data skew. Mean number of GIS data was not skewed

## Discussion

This study assessed the relationship between pre-competition psychological state and GIS during a 56 km ultramarathon running race and investigated sex differences and predictors of race GIS. Findings from this study show that psychological state across the three days prior to the race was correlated with race GIS. Another key finding was that pre-competition stress, anxiety, recovery, body mass and prior GIS predicted between 36 and 40% of variance in race GIS (number and severity). The severity of race GIS positively correlated with time to race completion, whereby a greater severity rating was associated with inferior race performance. Together, these findings suggest that pre-competition psychological state may be a contributor to GIS, and subsequently performance, during ultramarathon running.

**Fig. 2:**
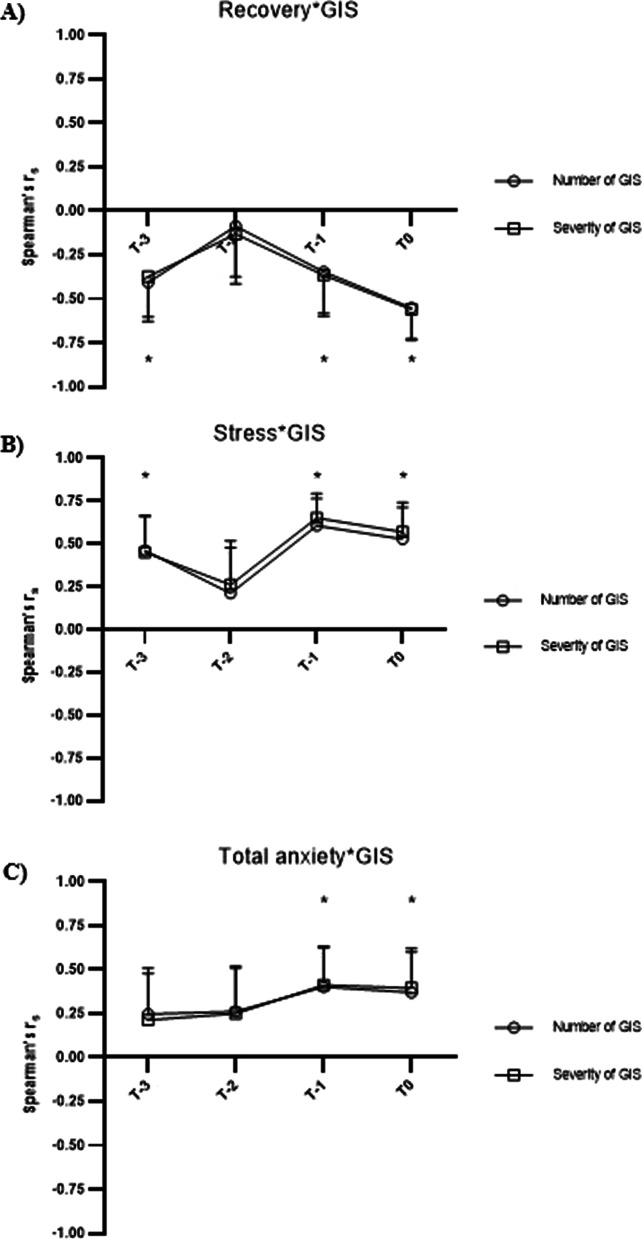
Correlation with gastrointestinal symptoms (GIS) in the days prior to a 56 km Ultramarathon running race. Correlation (Spearman’s rho) between recovery (**A**), stress (**B**) and total anxiety (**C**) with number and severity of GIS over the 3 days prior to (T-3, T-2, T-1) and on the morning of (T0) a 56 km Ultramarathon running race (all *n* = 44). *Statistically significant correlation between both number and severity of GIS with the relevant variable (*p* < 0.05)

In the current study, the number and severity of race GIS were positively correlated with pre-race stress and anxiety and negatively correlated with recovery, whereby participants that reported greater stress, greater anxiety or lower recovery prior to the ultramarathon also reported greater GIS during the race. The correlation between anxiety and GIS appeared to be strongest in the 24 h prior to the event (T-1 and T0, Fig. 2C), though the patterns for recovery and stress were less clear. The findings regarding stress and anxiety align with other recent studies that have identified that acute and chronic anxiety, and chronic stress are linked to GIS in training for endurance running and in endurance competition [9–11]. Weak to moderate correlations (*r*_s_ between 0.1 and 0.6 [27]) are often reported in studies examining predictors of GIS [9, 10, 28, 29], whereby the multifactorial causality of GIS in athletes may limit the extent to which any one factor can predict GIS. To the authors’ knowledge, this is the first study to identify a negative correlation between recovery and GIS. It is premature to suggest a causal relationship between these variables, but this presents an avenue for future research. Together, these findings suggest a potential interaction between psychological state and GIS in endurance runners during training and competition. Considering the bi-directionality (e.g. two-way relationship) between psychological state and GIS [30, 31], it is currently unknown if elevated stress/anxiety and reduced recovery contribute to GIS, or if GIS contributes to increased stress/anxiety and reduced recovery in endurance runners. The recruitment of runners free from gastrointestinal conditions, low GIS reported over the pre-race period and data from the regression analysis in this study suggest that psychological state may contribute to GIS. Elevated stress and/or anxiety has been proposed to contribute to GIS via altered corticotropin releasing factor (CRF) secretion that subsequently reduces splanchnic blood flow and/or alters gastrointestinal functions (e.g. decreases gastric emptying and motility and increases large intestine motility) [8]. Future research should aim to determine the direction and causation of the link between psychological state and GIS in endurance runners and use physiological markers (e.g. CRF, gastric emptying and motility, etc.) to determine the associated mechanisms. Future research is also required to determine the efficacy of strategies such as meditation and progressive muscle relaxation that can attenuate cognitive [32] and somatic anxiety [33], on GIS in endurance runners.

Particularly novel within this study was examining the extent to which GIS during the ultramarathon could be predicted by pre-race psychological state and prior GIS. Variation in the number and severity of GIS during the race was ascribed to changes in pre-race stress, anxiety, recovery, body mass and prior GIS. While each of these variables was included within the regression due to their strength of correlation with race GIS, body mass and prior GIS were the only variables to reach statistical significance (all *p* < 0.02, Table 3), thus contributing to this prediction to a greater extent than the psychological state variables. These findings are partially supported by previous research that has identified associations between history of prior GIS [17] and GIS during exercise in endurance athletes. Despite the association between body mass and GIS, body mass index (BMI) was not significantly correlated with GIS in the current study, contrasting with a prior investigation [6]. These differences between studies may have arisen due to study design considerations, such as selection of GIS assessment tools, or recruitment of both male and female participants in the current study. The apparent inconsistency between body mass and BMI in terms of impact on GIS may have arisen due to the mathematical nature of these two anthropometric measurements, whereby a change in body composition (e.g. increased fat mass) affects a larger relative change in body mass than in BMI, potentially dulling variation in the latter. Considering the multifactorial causes of GIS at rest and during exercise, athletes presenting a history of GIS should be advised to seek medical assessment for potential underlying pathophysiology, and further assessment of nutrition and exercise-related causes if required. The link between body mass or body mass index and GIS in endurance athletes is unclear, but could potentially be attributed to higher body fat [6] (e.g. more unfavourable body composition) and/or greater exercise stress associated with the weight-bearing characteristics of running. Indeed, the exercise undertaken by participants of the current study (56 km running) likely contributed to GIS [2, 16], represented by the large increase in number and severity of GIS from pre-race (at rest) to during the race, as seen in Additional file 1: Fig. S1. These findings point to the multifactorial causality of GIS during endurance exercise, in that participant and exercise characteristics as well as prior GIS and psychological state contribute to subsequent GIS during exercise.

Participant age did not significantly correlate with the number of severity of race GIS, in contrast to previous investigations, which reported an inverse relationship between the two (i.e. increased GIS with lower age) [6, 9]. This inconsistency may have arisen due to the vastly different exercise stimuli in each respective study, though this requires further investigation. A weak but statistically significant correlation was identified between the severity of race GIS and time to race completion, suggesting those with higher severity of GIS had slower race completion times. While this correlation does not suggest causation, the occurrence of GIS has previously been shown to impair exercise performance [2, 4] and has been identified as a major performance limiting factor in ultramarathon running [3]. Identifying factors that contribute to GIS and subsequent strategies to attenuate these factors are therefore important to athletes, coaches and sports practitioners. Considering the emerging evidence from this study and previous research, future studies should aim to investigate the effectiveness of strategies that can attenuate excessive psychological stress and anxiety in athletes that have a history of GIS.

Correlations between psychological state and GIS were stronger in male than female runners, despite similar total GIS values (number and severity) being reported across sexes and greater anxiety reported by females. While these differences may have been partially contributed to by the relatively small sample of female participants (18 participants), it is worth examining these sex-specific outcomes. The apparent sex difference may be a result of differences in the gut-brain axis function in males and females which have been proposed to arise due to the effect of circulating oestrogen on gastrointestinal motility [18], or may have resulted from other confounding factors (e.g. exercise-related, nutrition intake, etc.) that have been associated with GIS in athletes. Considering that female athletes often report greater anxiety and GIS than male athletes [16, 17, 19], future research is warranted to explore potential sex differences in the gut-brain function of athletes in response to exercise. Such research would allow the identification of targeted, and potentially sex-specific, strategies to attenuate excessive stress/anxiety and GIS in athletes.

The authors acknowledge the presence of several limitations within the current study. A relatively small sample size of 44 healthy recreationally active athletes limits the analysis and conclusions that can be made from this research, and the transferability of these findings to elite athletes. Future investigations should assess the interplay between psychological state and GIS in an elite population where competitive stress/anxiety is likely to be higher, and in athletes who have gastrointestinal conditions/disorders (e.g. IBS, inflammatory bowel disease, etc.) where the incidence of GIS is likely to be higher. Psychological state and GIS were assessed using subjective measurements only (i.e. surveys, questionnaires), so future investigations may consider monitoring physiological responses (e.g. CRF for GIS [8], heart rate variability for psychological state [34, 35]) that represent the subjective outcomes reported within the current study.

## Conclusion

In conclusion, psychological state (stress and anxiety) in the three days prior to a 56 km ultramarathon running race was associated with race GIS, and when combined with body mass and prior GIS accounted for 36–40% of the variation in race GIS. Pre-competition psychological state should be considered a potential contributor to GIS in ultramarathon alongside established factors such as exercise stress and nutrition intake. Further research is warranted to determine if modification of pre-competition psychological state can attenuate severe GIS and the associated decrement in race performance.

## Practical Applications

Based on the findings of the current study, the following can be recommended to athletes, and sports practitioners assisting athletes, competing in endurance exercise, such as ultramarathon running:

- Monitor stress, anxiety and GIS using reliable and valid questionnaires over the three days prior to competition.
- If GIS occur alongside elevated anxiety and/or stress, consider the implementation of targeted strategies to attenuate excessive stress or anxiety.
- If GIS occur in the absence of elevated stress and/or anxiety or if athletes present with a history of prior GIS, consider medical pathophysiology and/or nutritional and exercise-stress related causes.

## Novelty Statement

This is the first study to monitor and explore acute changes in psychological state and the relationship with GIS over the three days prior to and during a competitive endurance event and investigate potential sex differences in the relationship between psychological state and GIS in athletes. No prior investigation has reported a predictive model (regression) for estimating the occurrence (number and severity) of GIS during an ultramarathon based on prior GIS, stress, anxiety, recovery and body mass.

## Supplementary Information

**Additional file 1:** Occurrence and incidence of gastrointestinal symptoms prior to and during a 56 km Ultramarathon running race; Summary statistics for the regressions predicting gastrointestinal symptoms (GIS) during a 56 km Ultramarathon running race; Gastrointestinal symptoms (GIS) in the days prior to, pre-race and during a 56 km Ultramarathon running race.

## Data Availability

The datasets generated and/or analysed during the current study are not publicly available due to the conditions of the ethical approval provided by the Deakin University Human Research Ethics Committee.

## Abbreviations

BMI: Body mass index
CI: Confidence intervals
CRF: Corticotropin releasing factor
CSAI-2: Competitive state anxiety inventory 2
GIS: Gastrointestinal symptoms
IBS: Irritable bowel syndrome
REDCap: Research electronic data capture
R_s_: Spearman’s rho
SRSS: Short recovery stress scale

## References

[1] Costa R, Snipe R, Kitic C, Gibson P (2017). Systematic review: exercise-induced gastrointestinal syndrome—implications for health and intestinal disease. Aliment Pharmacol Ther.

[2] Stuempfle KJ, Hoffman MD (2015). Gastrointestinal distress is common during a 161-km ultramarathon. J Sports Sci.

[3] Hoffman M, Fogard K (2011). Factors related to successful completion of a 161-km ultramarathon. Int J Sports Physiol Perform.

[4] de Oliveira E, Burini R, Jeukendrup A (2014). Gastrointestinal complaints during exercise: prevalence, etiology, and nutritional recommendations. Sports Med.

[5] Wilson PB (2020). The psychobiological etiology of gastrointestinal distress in sport: a review. J Clin Gastroenterol.

[6] Ten Haaf DS, van der Worp MP, Groenewoud HM, Leij-Halfwerk S, Nijhuis-van der Sanden MW, Verbeek AL (2014). Nutritional indicators for gastrointestinal symptoms in female runners: The ‘Marikenloop study’. BMJ Open.

[7] Mikocka-Walus A, Ford AC, Drossman DA (2020). Antidepressants in inflammatory bowel disease. Nat Rev Gastroenterol Hepatol.

[8] Taché Y, Martinez V, Million M, Wang L (2001). Stress-related alterations of gut motor function: role of brain corticotropin-releasing factor receptors. Am J Physiol Gastrointest Liver Physiol.

[9] Wilson PB (2018). Perceived life stress and anxiety correlate with chronic gastrointestinal symptoms in runners. J Sports Sci.

[10] Wilson PB, Russell H, Pugh J (2021). Anxiety may be a risk factor for experiencing gastrointestinal symptoms during endurance races: an observational study. Eur J Sport Sci.

[11] Wilson PB (2020). Associations between sleep and in-race gastrointestinal symptoms: an observational study of running and triathlon race competitors. Sleep Sci.

[12] Li X, Kan E, Lu J, Cao Y, Wong R, Keshavarzian A (2013). Combat-training increases intestinal permeability, immune activation and gastrointestinal symptoms in soldiers. Aliment Pharmacol Ther.

[13] Worobetz L, Gerrard D (1985). Gastrointestinal symptoms during exercise in Enduro athletes: prevalence and speculations on the aetiology. N Z Med J.

[14] Sullivan SN (1987). Exercise-associated symptoms in triathletes. Phys Sports Med.

[15] Saw AE, Kellmann M, Main LC, Gastin PB (2017). Athlete self-report measures in research and practice: considerations for the discerning reader and fastidious practitioner. Int J Sports Physiol Perform.

[16] Keeffe EB, Lowe DK, Goss JR, Wayne R (1984). Gastrointestinal symptoms of marathon runners. West J Med.

[17] Peters H, Bos M, Seebregts L, Akkermans L, van Berge HG, Bol E (1999). Gastrointestinal symptoms in long-distance runners, cyclists, and triathletes: prevalence, medication, and etiology. Am J Gastroenterol.

[18] Pugh JN, Lydon K, O’Donovan CM, O’Sullivan O, Madigan S (2021). More than a gut feeling: what is the role of the gastrointestinal tract in female athlete health?. Eur J Sport Sci.

[19] Rice SM, Gwyther K, Santesteban-Echarri O, Baron D, Gorczynski P, Gouttebarge V (2019). Determinants of anxiety in elite athletes: a systematic review and meta-analysis. Br J Sports Med.

[20] Harris PA, Taylor R, Thielke R, Payne J, Gonzalez N, Conde JG (2009). Research electronic data capture (REDCap): a metadata-driven methodology and workflow process for providing translational research informatics support. J Biomed Inform.

[21] Martens R, Burton D, Vealey RS, Bump LA, Smith DE. Development and validation of the competitive state anxiety inventory-2. Compet Anxiety Sport. 1990;117–90.

[22] Nässi A, Ferrauti A, Meyer T, Pfeiffer M, Kellmann M (2017). Development of two short measures for recovery and stress in sport. Eur J Sport Sci.

[23] Gaskell SK, Snipe RMJ, Costa RJS (2019). Test–retest reliability of a modified visual analog scale assessment tool for determining incidence and severity of gastrointestinal symptoms in response to exercise stress. Int J Sport Nutr Exerc Metab.

[24] Kellmann M, Bertollo M, Bosquet L, Brink M, Coutts AJ, Duffield R (2018). Recovery and performance in sport: consensus statement. Int J Sports Physiol Perform.

[25] Kölling S, Schaffran P, Bibbey A, Drew M, Raysmith B, Nässi A (2020). Validation of the Acute Recovery and Stress Scale (ARSS) and the Short Recovery and Stress Scale (SRSS) in three English-speaking regions. J Sports Sci.

[26] Blake MR, Raker JM, Whelan K (2016). Validity and reliability of the Bristol Stool Form Scale in healthy adults and patients with diarrhoea-predominant irritable bowel syndrome. Aliment Pharmacol Ther.

[27] Dancey CP, Reidy J (2007). Statistics without maths for psychology.

[28] Pfeiffer B, Stellingwerff T, Hodgson AB, Randell R, Pöttgen K, Res P (2012). Nutritional intake and gastrointestinal problems during competitive endurance events. Med Sci Sports Exerc.

[29] Pfeiffer B, Cotterill A, Grathwohl D, Stellingwerff T, Jeukendrup AE (2009). The effect of carbohydrate gels on gastrointestinal tolerance during a 16-km run. Int J Sport Nutr Exe Metabol.

[30] Gracie DJ, Guthrie EA, Hamlin PJ, Ford AC (2018). Bi-directionality of brain–gut interactions in patients with inflammatory bowel disease. Gastroenterology.

[31] Koloski NA, Jones M, Kalantar J, Weltman M, Zaguirre J, Talley N (2012). The brain–gut pathway in functional gastrointestinal disorders is bidirectional: a 12-year prospective population-based study. Gut.

[32] Colzato LS, Kibele A (2017). How different types of meditation can enhance athletic performance depending on the specific sport skills. J Cognit Enhanc.

[33] Parnabas VA, Mahamood Y, Parnabas J, Abdullah NM (2014). The relationship between relaxation techniques and sport performance. Univ J Psychol.

[34] Chalmers JA, Quintana DS, Abbott MJ-A, Kemp AH. Anxiety disorders are associated with reduced heart rate variability: a meta-analysis. Front Psychiatry. 2014;5(80). 10.3389/fpsyt.2014.00080 PMC4092363 25071612

[35] Dishman RK, Nakamura Y, Garcia ME, Thompson RW, Dunn AL, Blair SN (2000). Heart rate variability, trait anxiety, and perceived stress among physically fit men and women. Int J Psychophysiol.

