# Supplementary material for: The Relationship Between Psychological Stress and Anxiety with Gastrointestinal Symptoms Before and During a 56 km Ultramarathon Running Race
Source: Sports Med Open. 2021 Dec 11;7:93. doi: 10.1186/s40798-021-00389-5 (PMC8665950; doi:10.1186/s40798-021-00389-5)
Supplement: Supplementary file 1 — Additional file 1: Occurrence and incidence of gastrointestinal symptoms prior to and during a 56 km Ultramarathon running race; Summary statistics for the regressions predicting gastrointestinal symptoms (GIS) during a 56 km Ultramarathon running race; Gastrointestinal symptoms (GIS) in the days prior to, pre-race and during a 56 km Ultramarathon running race. [file 40798_2021_389_MOESM1_ESM.docx]

**The relationship between psychological stress and anxiety with gastrointestinal symptoms before and during a 56km Ultramarathon running race.**

**Sports Medicine - Open**

Charles S. Urwin^1^, Luana C. Main^2^, Antonina Mikocka-Walus^3^, David R. Skvarc^3^, Spencer S.H. Roberts^2^, Dominique Condo^1^, Amelia J. Carr^1^, Lilia Convit^1^, William Jardine^1^, Shant S. Rahman^1^, Rhiannon M.J. Snipe^1^.

^1^ Deakin University, Melbourne, Victoria, Australia. Centre for Sport Research, School of Exercise and Nutrition Sciences.

^2^ Deakin University, Melbourne, Victoria, Australia. Institute for Physical Activity and Nutrition, School of Exercise and Nutrition Sciences.

^3^ Deakin University, Melbourne, Victoria, Australia. Centre for Social & Early Emotional Development, School of Psychology.

**ORCID iDs**

C.S.U – 0000-0002-9467-0077, L.C.M – 0000-0002-9576-9466, A.M.W – 0000-0003-4864-3956, D.R.S – 0000-0002-3334-4980, L.C – 0000-0001-5656-9106, S.S.R – N/A, W.J – 0000-0002-6323-0626, S.S.H.R – 0000-0002-9095-7070, D.C – N/A, A.J.C – 0000-0003-3855-2540, R.M.J.S – 0000-0002-3754-6782.

**Corresponding Author:**

Mr Charles Urwin

Email: [urwinc@deakin.edu.au](mailto:urwinc@deakin.edu.au)

**Additional file 1: Table S1.** Occurrence and incidence of gastrointestinal symptoms prior to and during a 56 km Ultramarathon running race.

|  | **Pre-race period occurrence** | **Pre-race period incidence (%)** | **Race occurrence** | **Race incidence (%)** |
| --- | --- | --- | --- | --- |
| **Upper Gastrointestinal Symptoms** | |  |  |  |
| Belching | 16 | 9 | 26 | 59 |
| Heartburn | 5 | 3 | 2 | 5 |
| Bloating | 32 | 18 | 12 | 27 |
| Stomach Pain | 10 | 6 | 15 | 34 |
| Urge to Regurgitate | 4 | 2 | 9 | 20 |
| Regurgitation | 2 | 1 | 5 | 11 |
| Vomiting | 1 | 1 | 1 | 2 |
| **Lower Gastrointestinal Symptoms** | |  |  |  |
| Flatulence | 74 | 42 | 27 | 61 |
| Intestinal Pain | 11 | 6 | 11 | 25 |
| Urge to Defecate | 28 | 16 | 19 | 43 |
| Constipation | 8 | 5 | 0 | 0 |
| Loose Stool | 37 | 21 | 5 | 11 |
| Diarrhoea | 6 | 3 | 1 | 2 |
| **Other Gastrointestinal Symptoms** | |  |  |  |
| Nausea | 4 | 2 | 9 | 20 |

Pre-race period includes all times prior to the commencement of the race (T-3, T-2, T-1, T0). Maximum value for the pre-race period occurrence of each symptom was 176 (44 participants reporting at 4 times). Maximum value for the race (TPost) occurrence of each symptom was 44 (44 participants reporting at 1 time). Pre-race period occurrence: number of times each symptom was reported before the race. Pre-race period incidence (%): percentage of pre-race time points where each symptom was reported. Race occurrence: number of participants that reported each symptom during the race. Race incidence (%): percentage of participants that reported each symptom during the race.

**Additional file 1: Table S2.** Summary statistics for the regressions predicting gastrointestinal symptoms (GIS) during a 56 km Ultramarathon running race.

| **Model** | **R Square** | **Adjusted R Square** | **Std. Error of the Estimate** | **F** | **df1** | **df2** | **Sig. F** |
| --- | --- | --- | --- | --- | --- | --- | --- |
| 1. Number of race GIS ^a^ | 0.470 | 0.400 | 1.867 | 6.730 | 5 | 38 | **0.000** |
| 2. Severity of race GIS ^b^ | 0.432 | 0.357 | 1.520 | 5.770 | 5 | 38 | **0.001** |

a. Predictors: (Constant), number of pre-race GIS, recovery, stress, total state anxiety, body mass. b. Predictors: (Constant), severity of pre-race GIS, recovery, stress, total state anxiety, body mass. Predictors included the sum of all pre-race times (T-3, T-2, T-1, T0). Bolded values indicate that the predictors in that model reliably predict changes in the dependent variable (*p* < 0.05). Note: a square-root transformation was performed for mean severity of GIS to account for data skew. Mean number of GIS data was not skewed.

**Additional file 1: Figure S1.** Gastrointestinal symptoms (GIS) in the days prior to, pre-race and during a 56 km Ultramarathon running race.


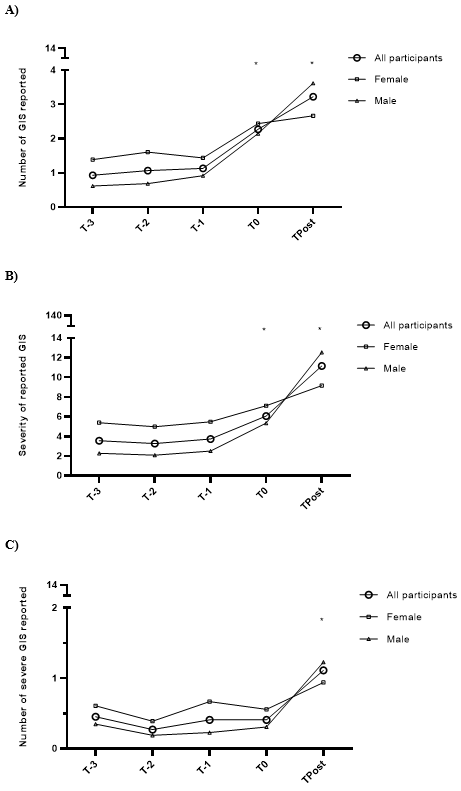


Mean (A) number of GIS reported, (B) severity of reported GIS, (C) number of severe GIS reported prior to (T-3, T-2, T-1, T0) and during (TPost) a 56 km Ultramarathon running race. GIS were classified as ‘severe’ when a participant rated the severity as ≥ 5 out of 10. All participants *n* = 44, female *n* = 18, male *n* = 26. ^*^ significantly higher rating compared to all prior time-points (*p* < 0.05), for all participants.
